# Impact of Weight Bias, Stigma and Discrimination on Physical, Mental, and Quality of Life Outcomes of Metabolic and Bariatric Surgery: A Systematic Review

**DOI:** 10.1007/s11695-026-08696-0

**Published:** 2026-05-02

**Authors:** Eugenia Romano, Ximena Ramos Salas, Lucia Alonso Diez, Ida Camperchioli, Chiara Gerardi, Violeta Moize, Ken Clare, Silvia Frusone, Francesco Maria Carrano

**Affiliations:** 1 King’s College London, London, UK; 2 Replica Communications, Kristianstad, Sweden; 3 European Association for the Study of Obesity, London, UK; 4 Hospital Clínic de Barcelona, Barcelona, Spain; 5 Hospital G.B. Grassi, Rome, Italy; 6 Mario Negri Institute for Pharmacological Research, Milan, Italy; 7 Obesity UK, Leeds, UK; 8 Sapienza University of Rome, Rome, Italy

**Keywords:** Weight stigma, Weight bias, Metabolic surgery, Bariatric surgery, Mental health, Quality of life, Weight management

## Abstract

**Background:**

Weight-related bias, stigma, and discrimination significantly affect quality of life and health in persons with obesity. Their influence on post-operative outcomes following metabolic surgery and bariatric remains underexplored.

**Objectives:**

This systematic review aimed to evaluate any impact of pre- and post-operative weight bias, stigma, and discrimination on post-metabolic and bariatric surgery outcomes, specifically physical health and mental health, including quality of life.

**Methods:**

This review was conducted in accordance with the PRISMA 2020 guidelines, with the protocol registered on PROSPERO. Comprehensive searches were performed across MEDLINE, PsycINFO, Embase, Web of Science, PEDro, CINAHL, ISRCTN, and CENTRA. Eligible studies included randomized controlled trials, clinical trials, longitudinal studies, cross-sectional studies, and qualitative research involving patients who underwent metabolic and bariatric surgery. Risk of bias was assessed using validated tools tailored to study design.

**Results:**

Eleven studies met the inclusion criteria, examining the influence of weight bias, stigma, and discrimination on post-surgical outcomes. Physical health outcomes included weight loss and BMI. Mental health outcomes included depressive symptoms, disordered eating behaviours, and quality of life domains such as social interactions, occupational settings, sexual health, educational experiences, and post-surgical health management. Findings suggest that weight bias negatively influences mental health and quality of life, associating with depressive symptoms, problematic eating, and lower adherence to nutritional instructions and exercise, potentially impeding optimal physical outcomes.

**Conclusions:**

Despite some studies suggesting its negative impact on postoperative outcomes, current evidence on the impact of weight bias, stigma, and discrimination on post-metabolic and bariatric surgery outcomes is limited. Critical gaps remain in understanding how these psychosocial factors affect long-term disease management, self-care, and overall quality of life.

**Supplementary Information:**

The online version contains supplementary material available at 10.1007/s11695-026-08696-0.

## Key Points

Some studies suggest that weight bias is associated with poorer weight change, worse mental health, and lower adherence to health behaviours in patients who underwent metabolic and bariatric surgery.

Psychosocial variables are still severely under-reported in patients who underwent metabolic and bariatric surgery.

Future approaches call for integrated care and tailored tools co-created with patients for the assessment of weight bias-related outcomes.

**Supplementary Information:**

The online version contains supplementary material available at 10.1007/s11695-026-08696-0.

## Introduction

Obesity is associated with poor mental health, including an increased risk for depression [1–3] and anxiety [4, 5], as well as disordered eating or substance use disorders [6–8]. Unfortunately, people living with obesity can also experience bias, stigma, and discrimination due to their weight [9], which can impact their quality of life [10, 11].

Weight bias refers to negative beliefs and attitudes about a person’s weight [12]. It is driven by social stereotypes that stipulate that people with obesity are lazy, unmotivated, and lack the willpower to manage their weight [12], and when an individual ends up applying those same believes to themselves because of their weight, then weight bias becomes internalized [13]; weight stigma is defined as the social devaluation and denigration of people perceived to carry excess weight and leads to prejudice, negative stereotyping and discrimination toward those people [14]; finally, weight discrimination is the unjust treatment of individuals because of their weight due to biased beliefs about weight and social sterotypes [15, 16]. People can internalize negative beliefs and stereotypes about weight and obesity, which can affect their health and well-being independently of their body mass index, weight, or obesity status [17–19], for example, leading them to avoid healthcare services for fear of being shamed and blamed for their weight by their healthcare providers [19, 20]; this is defined as weight shame, which is an attributed (external) or self-perceived (internal) sense of inferiority or devaluation because of one’s own weight [21] Weight stigma can also be a barrier for self-care and health promoting behaviors such as physical activity [22] and healthy eating [23, 24]. Furthermore, people living with obesity who internalize weight-related prejudices may blame themselves for their condition, further worsening their health [25].

The pervasive social stigma associated with obesity [19, 26, 27] can also trigger physiological and behavioral responses in people living with obesity, which, in turn, will worsen their condition, for example by causing heightened levels of cortisol which would induce further weight gain and increased hunger, leading to overeating patterns [14, 19, 28]. Weight-based discrimination has also been associated with increased mortality, independent of other physical and mental health factors, therefore being a social determinant of health [19, 29]. Finally, people living with obesity may also experience weight-based discrimination in education or employment settings, with barriers to accessing higher-income jobs, inequities in wages, and a lower chance of obtaining higher education [30–32].

Studies have shown that some patients living with obesity expect less weight stigma experiences following their obesity treatments, but this is not always the case [9, 33–36]. There is evidence that stigma doesn’t end after metabolic and bariatric surgery, showing its pervasiveness [9], with patients frequently encountering discriminatory healthcare interactions which may contribute to weight regain, poor mental health, and poor healthcare engagement [35]. This, paired with the stigma posed on patients who undergo metabolic and bariatric surgery for ‘choosing the easy way out’ [37, 38], could impact different post-operative outcomes, hindering the improvements associated with patients seeking bariatric and metabolic surgery. Furthermore, the impact of preoperative weight bias, stigma, and discrimination on physical and psychological health after metabolic and bariatric surgery is unclear, as well as its effect on quality of life.

Although the impact of weight stigma around metabolic and bariatric surgery has been reviewed [39], no study so far has focused on summarizing post-surgery outcomes, including qualitative results. Thus, this review aimed to be the first to systematically provide information on the impact of pre- and post-operative weight bias, weight stigma (both internalized and external) and/or weight discrimination on three main postoperative domains in adult patients living with obesity who underwent metabolic and bariatric surgery, considering physical outcomes (such as weight change, metabolic rates etc.) and mental health outcomes (such as depressive symptoms, anxiety, eating disorders etc.), including various aspects of quality of life, such as relationships, work satisfaction, or health-related behaviours and health and weight-related management.

## Methods

The systematic review protocol of this study was registered with the International Prospective Register of Systematic Reviews (PROSPERO). The Preferred Reporting Items for Systematic Reviews and Meta-Analyses (PRISMA) 2020 guidelines have been followed for reporting the review [40]. Participants were included if they underwent metabolic and bariatric surgery, Roux-en-Y gastric bypass, sleeve gastrectomy, gastric banding, single anastomosis duodenal-ileal bypass with sleeve gastrectomy, one anastomosis gastric bypass, or biliopancreatic diversion, with a body mass index (BMI) ≥ 25 at the time of intervention and older than 18 years of age, from all ethnic groups. Studies published in English were considered, with designs including randomized controlled trials, clinical trials, longitudinal studies, cross-sectional studies, and qualitative studies, in case any of these reported an evaluation of weight stigma, bias or discrimination in the pre- or post- assessments, while they were excluded in case there was no measure of stigma or if the design did not match our terms of search (e.g. stigma measures, but study not exploring metabolic and bariatric surgery). Cross-referencing was also used by checking references of included studies to identify additional relevant articles.

An experienced researcher and methodology expert was consulted on the methodology. A medical subject heading (MeSH) analysis of known key articles provided by the research team was performed. MEDLINE, PsycINFO, Embase, Web of Science, PEDro, CINAHL, ISRCTN, and CENTRAL databases were systematically searched from inception until July 31 st, 2023, for pertinent literature. The search strategy is available in supplementary material. A second search was conducted using the same search strings but considering only manuscripts published from July 31 st, 2023, to February 22nd, 2024, to check for any new relevant titles, and again a third one, from February 22nd 2024 to September 3rd 2025, and a fourth one, from September 3rd 2025 to March 24th 2026.

### Study Selection

To streamline the initial screening of abstracts and titles resulting from the search, the authors utilized Rayyan (http://rayyan.qcri.org), a software harnessing natural language processing (NLP) and machine learning (ML) technologies tailored for systematic reviews, allowing to filter studies based on predefined lists of keywords, assign labels to citations, and establish reasons for exclusion. Rayyan has been proved a reliable tool for the practice of systematic reviews [41].

### Data of Interest and Data Extraction

All the authors who reviewed titles and abstracts have background in obesity as either researchers or healthcare professionals. Each title and abstract were reviewed by at least two reviewers working independently; final decisions on disagreements were made by a third independent reviewer. Reviewers agreed on 86% of evaluations (with a minimum of 80% required as substantial [42]).

**Fig. 1:**
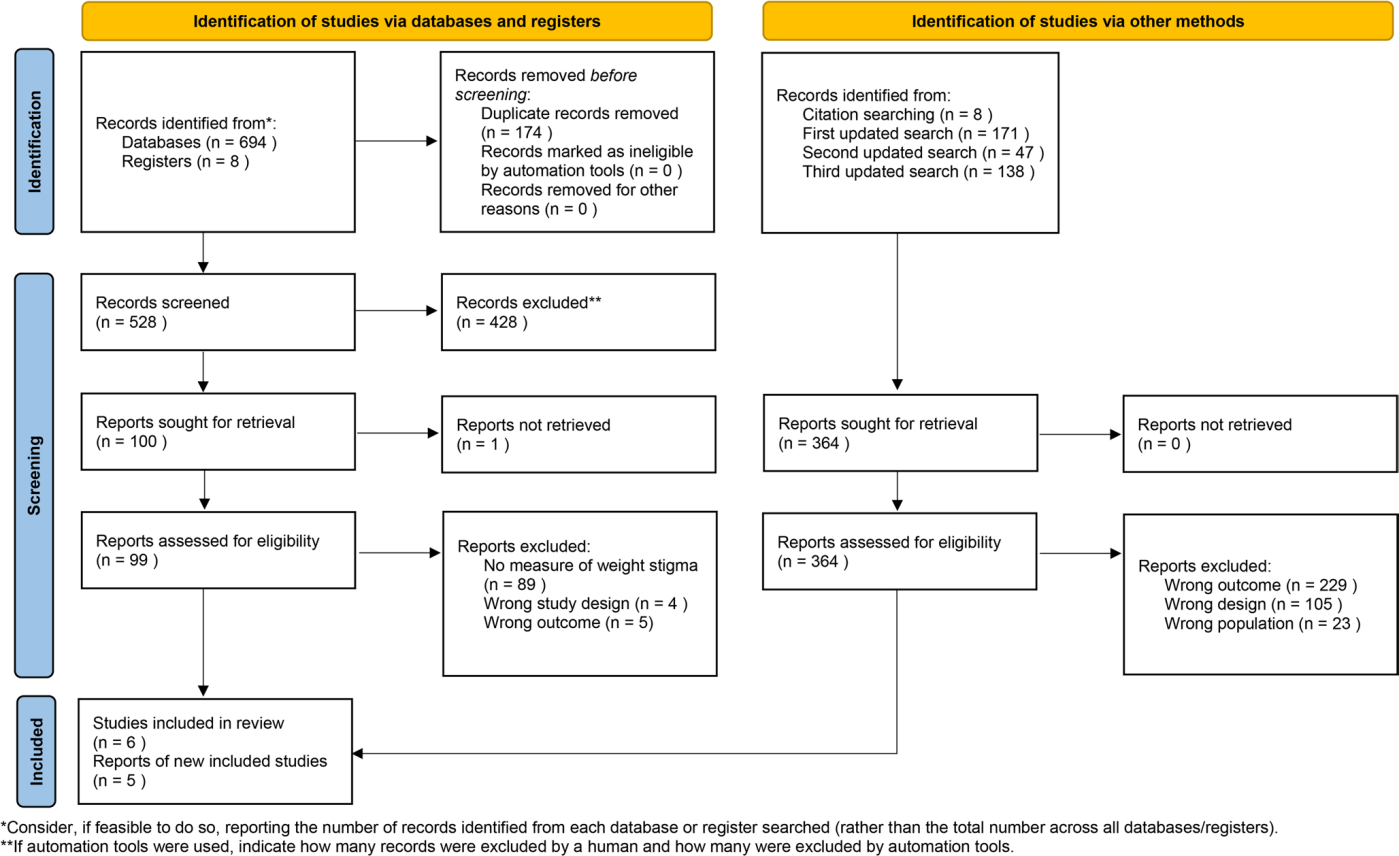
PRISMA checklist for identified and included studies. Source: Page MJ, et al. BMJ 2021;372:n71. doi: 10.1136/bmj.n71. This work is licensed under CC BY 4.0. To view a copy of this license, visit https://creativecommons.org/licenses/by/4.0/

Data from included studies were extracted by a single reviewer and then verified by a second one using a standardized form developed for the review, including items related to publication year and country, study desig using the Newcastle-Ottawa Scale for cohn, study settings, information on participants, the nature of the intervention, follow-up details, primary and secondary outcomes, any exclusions of participants, and the reasons for these, confounders, and risk of bias. A statistical synthesis of the associations between weight bias, weight stigma, or weight discrimination and measured outcomes was planned in case enough data were available. All extracted data was recorded in an Excel file. This file will be available as a supplementary appendix. Meta-analysis was not performed due to heterogeneity of results. PRISMA checklist is reported in Fig. 1.

### Risk of Bias and Quality of Studies Assessment

A risk of bias assessment of the included articles was carried out using the Newcastle-Ottawa Scale for cohort studies [43]. The Version 2 of the Cochrane risk-of-bias tool for randomized trials (RoB2) [44] was used to inform the Grading of Recommendations, Assessment, Development, and Evaluations (GRADE) [45] tool that is used to assess the quality of evidence. To assess quality of cross-sectional studies, the JBI Critical Appraisal Checklist for Analytical Cross-Sectional Studies [46] was employed. Quality appraisal of qualitative studies was conducted using the Critical Appraisals Skills Programme (CASP) tool for qualitative research [47]. Two studies [36, 48] were rated as high quality, while the remaining five [49–53] were assessed as moderate due to limitations such as lack of control groups or limited confounder adjustment.

## Results

### Study Characteristics

Eleven studies met the inclusion criteria, comprising eight quantitative studies, two qualitative study, and one mixed-methods design [36, 48–57]. Together, these involved a total of 2,231 participants, with sample sizes ranging from 14 to 564. Most studies were conducted in the USA (*n* = 5), while others were based in Europe, including Poland, Germany, Sweden, and the Netherlands. Across studies that reported gender, most participants were female (ranging from 75% to 100%). Preoperative BMI across studies ranged from 41.8 [55] to 47.8 [50]. Postoperative BMI varied substantially across studies, ranging from BMI values of approximately 30 kg/m² [54, 56] to post-surgical BMI ranges of 16–70 kg/m² [52]. Study quality was judged moderate in nine studies and high in two qualitative or mixed-methods studies. Results (as well as original extraction sheet) are reported in Table 1.

**Table 1:** Summary of included studies

Study	Geographical region	Participants	Gender	BMI	Type of Study	Results	Quality of evidence
Hoffmann et al. (2022)	Poland and Germany	564 adult participants	76.6% female	BMI between 34.67 ± 8.89 and 37.46 ± 9.95	Quantitative, retrospective cross sectional international multicenter survey study	Mental Health and Quality of Life: last 6-months experienced postoperative discrimination reported no significant associations with levels of education.	Moderate (Single-cohort design, limited confounder control)
Feig et al. (2020)	Not specified	112 adults	91.1% female	Mean BMI 31.3 ± 7.4	Quantitative, Cross-sectional study	Physical Health: postoperative higher internalised weight bias was associated with lower BMI change (*b* = −1.14, *p* =.04), internalised weight bias was positively associated with BMI (*b* 0.08, *p* <.001) Mental Health and Quality of Life: postoperative higher internalised bias associated with poorer moderate-to-vigorous physical activity via indirect effect through increased depression (0.44, SE = 0.14, bias-corrected 95% CI: −0.78 to − 0.20). Postoperative higher internalised stigma associated with poorer adherence to health behaviors such as diet (*b* − 0.85, *p* <.01) and supplementation adherence (*b* − 0.36, *p* 0.04), more barriers to being active (*b* 3.16, *p* <.01) and lower self-efficacy for exercise (*b* −4.67, *p* <.01), lower moderate-to-vigorous physical activity (*b* − 0.67, *p* <.05), and with lower mental health-related quality of life (*b* −3.77, *p* <.001). Indirect effect of postoperative internalised weight bias on poorer moderate-to-vigorous physical activity through lower self-efficacy for exercise was − 0.23 (SE = 0.12, bias-corrected 95% CI: −0.51 to − 0.05), while through increased barriers to being active was − 0.35 (SE = 0.19, bias-corrected 95% CI: −0.62 to − 0.13).	Moderate (No comparison group, incomplete confounder adjustment)
Lent et al. (2014)	USA	170 adults	81.9% female	Mean BMI was 47.8 ± 8.3 kg/m2 pre-surgery and 32.5 ± 6.1 kg/m2 at 12-months post-surgery	Quantitative, Prospective observational cohort study	Physical Health: greater preoperative internalised weight bias was associated with less weight loss 1 year after surgery (B = − 1.41, *p* 0.035). Mental Health: greater preoperative internalised weight bias was associated with more depressive symptoms before surgery (B = 3.66, *p* <.001)	Moderate (No comparison group, moderate follow-up)
Raves et al. (2016)	USA	300 postoperative bariatric patients	N/A	N/A	Mixed methods, observational study + thematic analysis	Physical Health: postoperative higher BMI associated with increased postoperative general (0.57, 95%CI 0.41; 0.73) and healthcare (0.1, 95%CI 0.09;0.17) weight-related stigma. Mental Health and Quality of Life: postoperative higher postoperative internalised bias (0.35, *p* <.01) and experienced stigma (0.16, *p* <.05) associated with higher disordered eating. Postoperative internalised bias inversely associated with dietary success (− 0.19, *p* <.05) and dietary adherence (− 0.18, *p* <.05). Qualitative reports of current clinic-based stigma leading to avoidance of follow-up care.	High (Potential selection bias due to low sample diversity, potential lack of reflexivity)
Tolvanen et al. (2021)	Sweden	16 participants	75% female	BMI range from 42 to 70	Qualitative study, semi-structured interviews	Mental Health and Quality of Life: postoperative fear of stigmatizing treatment was a barrier to social activities. Postoperative shame of being perceived as the heaviest one (internalised bias) impacted participating in an obesity treatment group. Negative comments from family or friends worsened guilt and self-esteem.	High (Potential selection bias due to low sample diversity, lack of details on reflexivity)
Han et al. (2018)	USA	298 participants	77% female	Post-surgical BMI from 16 to 70	Quantitative, structural equation modelling	Mental Health and Quality of Life: higher experienced weight stigma post-surgery (0.008, 95% CI 0.001; 0.030) and post-surgery internalised weight bias 0.066, 95%CI 0.013; 0.190) negatively impacted physical activity via the effect of increased exercise avoidance (direct effects on exercise avoidance 0.303, *p* <.05 and 240, *p* <.01 respectively)	Moderate (Potential reporting bias, lack of comparison group)
Konings et al. (2023)	Netherlands	101 participants	N/A	Initial mean weight 134.5 kg. (SD: 24.1)	Quantitative, logistic regression and t-tests	Physical Health: participants reporting no improvement in preoperative shame (used as a subjectively reported psychosocial complaints related to obesity and its treatment using a validated scale) after the surgical intervention showed less weight loss than those who reported shame as ‘resolved’ (16.4 kg, SD:20.0 versus 46.1 kg, SD: 14.0; *p* <.001). Feelings of shame improved post-surgery, but minimal change was associated with less weight loss.	Moderate (Lack of control group, limited control for confounding variables)
McGarrity et al., 2025	USA	148	81% female	Initial mean BMI: 44.98 kg/m² (SD: 6.52)	Quantitative: regression analysis	Physical health: Experienced weight stigma (EWS) was significantly associated with weight-related outcomes both before and after bariatric surgery. At baseline, higher EWS was moderately correlated with higher BMI (*r* = −.53, *p* <.001), and this association strengthened post-surgery (*r* = −.63, *p* <.001). Reductions in EWS from pre- to post-surgery were significantly associated with reductions in BMI (*r* = −.42, *p* <.001). In multivariable regression analyses controlling for baseline BMI and sociodemographic factors, greater improvement in EWS predicted lower post-surgical BMI (B = − 0.09, *p* <.001). Similarly, higher post-surgical EWS independently predicted higher BMI (B = − 0.14, *p* <.001). Mental health and Quality of Life: post-surgery, higher EWS was significantly associated with worse mental health and behavioural outcomes, including depression (*r* = −.36), anxiety (*r* = −.30), binge eating (*r* = −.34), and disordered eating (*r* = −.34) (all *p* <.05). Pre to post-surgery improvements in EWS were significantly correlated with improvements in depression (*r* = −.35, *p* <.001), anxiety (*r* = −.17, *p* =.04), binge eating (*r* = −.30, *p* <.001), and disordered eating (*r* = −.37, *p* <.001). In adjusted regression models, greater reductions in EWS predicted lower post-surgical depressive symptoms (B = − 0.07, *p* <.001), anxiety (B = − 0.04, *p* <.05), binge eating (B = − 0.07, *p* <.01), and disordered eating (B = − 0.02, *p* <.001). Higher levels of post-surgical EWS remained independently associated with worse outcomes across depression (B = − 0.09, *p* <.001), anxiety (B = − 0.07, *p* <.05), binge eating (B = − 0.12, *p* <.001), and disordered eating (B = − 0.02, *p* <.001), even after controlling for baseline measures and BMI.	Moderate (attrition between baseline and follow-up, reliance on self-reported post-surgical BMI)
Pan et al., 2026	China	452	64.5% female	Mean pre-surgery BMI: 41.80 kg/m² (SD 7.46)	Quantitative: correlations, multivariate regression	Physical health: preoperative internalized weight bias was associated with weight change among female participants (*r* =.151, *p* =.007), but association was non significant in multivariate regression (B:0.079, 95% CI: −0.031—0.132, p: 0.225)	Moderate (low generalizability, reliance on self-reported psychological measures)
Dunford et al., 2025	USA	56	93% female	Average presurgical BMI: 47.6 kg/m2 (SD 8.45); average Postsurgical (average 39.5 months since intervention) BMI: 30.1 kg/m2 (SD 5.3) Average percent total weight loss since surgery: 36% (SD 8.95)	Quantitative: linear mixed models	Mental Health and Quality of Life: postoperative weight bias internalization was positively associated with eating-disorder psychopathology and depression at three different time points after body contouring: baseline (*r* >.40, *p* <.01), 1-month (*r* >.33, *p* <.01), and 3-month (*r* >.45, *p* <.01) follow-ups.	Moderate (small, predominantly female sample, restriction to patients seeking body contouring surgery, and reliance on self-reported measures)
Garcia et al., 2026	Netherlands	14	100% female	Mean BMI ≈ 29.7 kg/m²	Qualitative: semi-structured interviews	Mental Health and Quality of Life: Internalised weight stigma was associated with relationships with food after the intervention, social interactions, self-acceptance and interactions with healthcare staff.	Moderate (small sample, potential selection bias)

### Physical Health Outcomes

Six studies investigated weight- or BMI-related outcomes [36, 49–51, 55, 57]. In a prospective cohort study [50], greater preoperative internalised weight bias predicted less weight loss at one year, while another study [51] showed that participants whose preoperative psychosocial complaint of shame remained unresolved after metabolic and bariatric surgery lost less weight than those who reported shame as resolved (16.4 kg vs. 46.1 kg; *p* <.001); cross-sectional analyses [49] found that higher postoperative internalised bias was associated with lower BMI change (b = − 1.14, *p* =.04) and with higher current BMI (b = 0.08, *p* < .001), while a mixed-methods study [36] further reported that current higher BMI was positively associated with both general and healthcare-related postoperative stigma. Finally, pre and post-surgical experiences of weight stigma [57] and preopertative internalised weight bias [55] were found to be significantly associated with weight-related outcomes both before and after bariatric surgery.

### Mental Health Outcomes and Quality of Life

Mental health outcomes were reported in nine studies [36, 48–50, 52–54, 56, 57]. Six of them evaluated depressive symptoms or disordered eating in relation to weight bias [36, 49, 50, 56, 57]. One [50] found that higher preoperative internalised bias was associated with more depressive symptoms (B = 3.66, *p* <.001), while in postoperative samples [49], postoperative internalised bias was indirectly linked to reduced physical activity via elevated depressive symptoms (B = 0.44, SE = 0.14, bias-corrected 95% CI: −0.78 to − 0.20); one study found higher experiences of weight stigma post-surgery to be significantly associated with depression (*r* = −.36) and anxiety (post-surgery: *r* = −.30), binge eating (*r* = −.30, *p* <.001), and disordered eating (*r* = −.37, *p* <.001), and that, in adjusted regression models, greater reductions in EWS predicted lower post-surgical depressive symptoms (B = − 0.07, *p* <.001), anxiety (B = − 0.04, *p* <.05), binge eating (B = − 0.07, *p* <.01), and disordered eating (B = − 0.02, *p*<.001) [57]. Another study reported postoperative weight bias internalization to be positively associated with depression at three different time points after post-surgical body contouring: baseline (*r* >.40, *p* <.01), 1-month (*r* >.33, *p* <.01), and 3-month (*r* >.45, *p* <.01) follow-ups [56]. A study [36] identified that both postoperative internalised bias (β = 0.35, *p* <.01) and experienced stigma (β = 0.16, *p* <.05) were positively associated with disordered eating behaviours. Finally, a qualitative study reported internalised weight stigma to be associated with relationships with food after the intervention [54].

Six studies reported on quality of life domains, including health behaviours, social relationships, and physical activity [36, 48, 49, 52–54]. One [49] showed postoperative internalised stigma was associated with lower diet adherence (b = − 0.85, *p* =.07), lower supplementation adherence (b = − 0.36, *p* =.04), more barriers to being active (b = 3.16, *p* <.01), and lower exercise self-efficacy (b = − 4.67, *p* <.05), as well as linked to lower moderate-to-vigorous physical activity (b = − 0.67, *p* <.05) and poorer mental health-related quality of life (b = − 3.77, *p*<.001). Another study [52] showed that higher postoperative experienced weight stigma (β = 0.008, 95% CI 0.001–0.030) and internalised weight bias (β = 0.066, 95% CI 0.013–0.190) were both associated with increased avoidance behaviours, which in turn negatively impacted physical activity; finally, qualitative evidence [48] showed that postoperative fear of stigmatizing treatment was a barrier to social activities and participation in group-based obesity treatment, while in healthcare settings [36], postoperative clinic-based stigma impaired trust in providers, with avoidance of follow-up care and reduced adherence to dietary recommendations, with some explicitly reporting avoidance of follow-up care. One cross-sectional study [53] however reported no association between postoperative perceived discrimination and educational attainment. Finally, a qualitative study reported internalised weight stigma to be associated with social interactions, self-acceptance and interactions with healthcare staff after the intervention [54].

## Discussion

This review aimed to systematically provide information on the impact of pre- and postoperative weight bias, weight discrimination, and weight stigma on three main postoperative domains (physical health and mental health, including quality of life) in adult patients with obesity who underwent metabolic and bariatric surgery, considering weight loss outcomes, mental health, and various aspects of quality of life, such as relationships and work satisfaction.

Only seven studies looking into weight discrimination, bias, and stigma in the context of post-bariatric outcomes were found. Overall, the evidence suggests a detrimental impact of weight bias, weight stigma and weight discrimination on BMI change, mental health, and health-related behaviours such as exercise avoidance, weight management and dietary adherence, as well as on social activities. However, none of these studies considered the impact of weight discrimination, stigma and bias on physical dimensions despite their well-known physiological impact on the organism [28, 58], which shows a potential underestimation of their detrimental effects. This is concerning, as weight bias, stigma and weight-based discrimination are considered important physical and mental health determinants for patients living with obesity [59], compromising their relationship with healthcare providers [20], affecting physical activity [22] and healthy eating [23, 24], as well as causing hormonal alteration [14, 28] and damaging their health [25]. Yet, according to the lack of studies in this systematic search, these measures are not consistently included in studies related to metabolic and bariatric surgery or randomized control trials, and are not incorporated in routine assessments of patients, both before and after the intervention, to evaluate their impact on outcomes related to physical (e.g. hormonal levels, cardiovascular profiles) and mental health (e.g., body image, body dissatisfaction, depressive symptoms, anxiety, disordered eating, eating disorders, and internalized weight bias) or social aspects (e.g. social exclusion, discrimination in education, workplaces, or healthcare settings) of patients’ lives, both on short and long term. These dimensions should be investigated in the future, as, according to this review, the type of stigma measured also appears to influence observed outcomes. Internalised weight bias appeared more consistently associated with psychological outcomes such as depression and disordered eating [49, 56, 57], whereas experienced stigma, particularly in healthcare settings, was more strongly linked to behavioural responses such as avoidance of care and reduced adherence to treatment recommendations [36, 52]. This suggests that different forms of stigma may operate through distinct mechanisms, with internalised weight bias primarily influencing psychological processes such as mood and eating behaviours, while experienced stigma may exert its effects indirectly through behavioural pathways, including disengagement from healthcare and reduced adherence to treatment recommendations.

The evidence collected by systematic review also suggests how the impact of weight bias, stigma, and discrimination differs across outcome domains. Across studies, associations with mental health outcomes and quality of life were relatively consistent, with higher levels of internalised or experienced stigma linked to greater depressive symptoms, disordered eating behaviours, reduced physical activity, and poorer adherence to lifestyle recommendations [36, 49, 52, 56, 57]. In contrast, associations with weight-related outcomes were less consistent. Some studies reported that higher preoperative or postoperative internalised weight bias was associated with poorer weight loss or higher postoperative BMI [49, 50, 57], whereas others found no independent association after adjustment for confounders [55]. This heterogeneity may reflect differences in study design, timing of assessment (pre- vs. post-operative), and the type of stigma measured (internalised vs. experienced), but it may also indicate how weight stigma influences physical outcomes indirectly, for example through behavioural pathways such as reduced adherence to dietary recommendations, avoidance of follow-up care, or lower self-efficacy for physical activity [36, 49, 52] rather than through direct physiological mechanisms. It is also true that we did not find many studies investigating a direct effect of stigma on physiological mechanisms in the population we investigated, which may be an indication for potential future studies.

These results call for more published evidence on the impact of weight stigma, weight bias and weight discrimination on postoperative dimensions of metabolic and bariatric surgery, possibly considering outcomes such as weight recurrence, long-term follow up, self-care, and health behavior change. For example, there is evidence that weight bias internalization can lead to patients avoiding healthcare services [20, 60, 61], which could be one reason for the significant loss to follow up in metabolic and bariatric surgery programs. Internalized weight bias can also impact chronic disease self-care management processes such as adherence to health behavior changes and regular follow up with healthcare providers [62, 63].

Inclusion of measures such as weight bias, stigma, and discrimination measures as part of metabolic and bariatric surgery procedure should become a key step to ensure the patient can truly benefit from the intervention. A recent study found that an internalized weight bias intervention prior to behavioral weight management intervention improved weight self-stigma, eating self-efficacy, and some aspects of quality of life [62]; similarly, assessment of weight stigma and weight bias-related dimensions in standard practice should become a mandatory step in metabolic and bariatric surgery practice. For example, healthcare professionals could examine the level of internalized weight bias among patients seeking metabolic and bariatric surgery, referring those with critical levels to psychological interventions prior to the intervention, to ensure better postoperative outcomes in the long-term and optimizing standards of care. Finally, future studies should also evaluate the impact of reduction interventions for weight stigma, weight bias and weight-based discrimination in metabolic and bariatric surgery, including policy interventions to prevent the unjust treatment of people living with obesity in workplaces and healthcare settings, and interventions for healthcare professionals to understand the link between weight bias, stigma, and weight-based discrimination on physical and mental health outcomes, as well as quality of life measures and healthcare delivery.

Overall, this study comes with significant strengths, such as a solid methodology (e.g. following PRISMA guidelines, being guided by a patients’ representative) and the input of a patient representative which made sure that the research goal was informed by stakeholders’ priorities. However, there are also significant limitations to address, such as the limitation to studies published in English. Another important point to underline is that some of the studies found did not have direct or explicit measures of weight bias, weight stigma, and weight-based discrimination, but relied on recalling of experiences without the use of a dedicated tool (e.g. the Stigmatizing Situations Inventory [38] or an edited version of the Weight Bias Internalization Scale [64]). Moreover, the fact that some titles had to be hand-searched also shows a potential inconsistency in the reporting of these themes in literature. To address this, future research should consider co-designing new and improved tools to assess the dimensions of weight stigma, weight bias and weight discrimination in collaboration with patients, to provide more consistent measurements and tackle those areas of impact relevant to the population of interest which are often under-assessed in metabolic and bariatric surgery practice.

## Conclusion

Although the evidence on weight stigma, bias, and discrimination clearly demonstrates their detrimental consequences for physical and mental health, their role in shaping outcomes after metabolic and bariatric surgery remains underexamined. The emerging data consistently point to adverse effects on postoperative weight loss, weight maintenance, adherence to lifestyle recommendations, and mental health status. These findings underline an urgent need to recognize and address weight-related psychosocial determinants as integral components of metabolic and bariatric care. Future interventions should incorporate systematic assessment of weight stigma, bias, and discrimination, using validated or patient co-designed instruments, to more accurately capture their influence on surgical trajectories. Embedding such measures into routine practice will not only enhance understanding of these factors but also enable targeted psychosocial support, ultimately improving long-term outcomes and ensuring equitable benefit from surgical treatment.

## Supplementary Information

Below is the link to the electronic supplementary material.

Supplementary Material 1 (DOCX 17.7 KB)

## Data Availability

No datasets were generated or analysed during the current study.

## References

[1] Luppino FS, De Wit LM, Bouvy PF, Stijnen T, Cuijpers P, Penninx BWJH, et al. Overweight, obesity, and depression: a systematic review and meta-analysis of longitudinal studies. Arch Gen Psychiatry. 2010;67(3):220–9. 10.1001/archgenpsychiatry.2010.2. 20194822 10.1001/archgenpsychiatry.2010.2

[2] Milaneschi Y, Simmons WK, Van Rossum EFC, Penninx BW. Depression and obesity: evidence of shared biological mechanisms. Mol Psychiatry. 2019;24(1):18–33. 10.1038/s41380-018-0017-5. 29453413 10.1038/s41380-018-0017-5

[3] Park SC, Kato TA, Lee JH, Yu SH. Exploring the bidirectional relationship between depression and obesity. Endocrinol Metab Clin North Am. 2025;54(1):193–206. 10.1016/j.ecl.2024.10.010. 39919875 10.1016/j.ecl.2024.10.010

[4] Amiri S, Behnezhad S. Obesity and anxiety symptoms: a systematic review and meta-analysis. Neuropsychiatr. 2019;33(2):72–89. 10.1007/s40211-019-0302-9. 30778841 10.1007/s40211-019-0302-9

[5] Sousa R, Esteves D, Sousa R, Machado J, Abelha A. Exploring the bidirectional relationship between obesity and mental health disorders. Procedia Comput Sci. 2025;257:1116–21. 10.1016/j.procs.2025.03.147.

[6] Golzarand M, Salari-Moghaddam A, Mirmiran P. Association between alcohol intake and overweight and obesity: a systematic review and dose-response meta-analysis of 127 observational studies. Crit Rev Food Sci Nutr. 2022;62(29):8078–98. 10.1080/10408398.2021.1925221. 33998940 10.1080/10408398.2021.1925221

[7] Wilson GT. Eating disorders, obesity and addiction. Eur Eat Disord Rev. 2010;18(5):341–51. 10.1002/erv.1048. 20821736 10.1002/erv.1048

[8] Yoon C, Mason SM, Hooper L, Eisenberg ME, Neumark-Sztainer D. Disordered eating behaviors and 15-year trajectories in body mass index: findings from Project Eating and Activity in Teens and Young Adults (EAT). J Adolesc Health. 2020;66(2):181–8. 10.1016/j.jadohealth.2019.08.012. 31630924 10.1016/j.jadohealth.2019.08.012 PMC6980455

[9] Brown A, Flint SW, Batterham RL. Pervasiveness, impact and implications of weight stigma. eClinicalMedicine. 2022;47:101408. 10.1016/j.eclinm.2022.101408. 35497065 10.1016/j.eclinm.2022.101408 PMC9046114

[10] Flint SW, Vázquez-Velázquez V, Le Brocq S, Brown A. The real‐life experiences of people living with overweight and obesity: a psychosocial perspective. Diabetes Obes Metab. 2025;27(S2):35–47. 10.1111/dom.16255. 39931901 10.1111/dom.16255 PMC12000856

[11] Latner JD, Barile JP, Durso LE, O’Brien KS. Weight and health-related quality of life: the moderating role of weight discrimination and internalized weight bias. Eat Behav. 2014;15(4):586–90. 10.1016/j.eatbeh.2014.08.014. 25215477 10.1016/j.eatbeh.2014.08.014

[12] Puhl R, Brownell KD. Bias, discrimination, and obesity. Obes Res. 2001;9(12):788–805. 10.1038/oby.2001.108. 11743063 10.1038/oby.2001.108

[13] Lay S, Birch R. Supporting patients with obesity. Practice Management. 2023;33(2):32–6. 10.12968/prma.2023.33.2.32.

[14] Tomiyama AJ. Weight stigma is stressful. A review of evidence for the cyclic Obesity/weight-based stigma model. Appetite. 2014;82:8–15. 10.1016/j.appet.2014.06.108. 24997407 10.1016/j.appet.2014.06.108

[15] Puhl RM, Latner JD, O’Brien KS, Luedicke J, Danielsdottir S, Salas XR. Potential policies and laws to prohibit weight discrimination: public views from 4 countries. Milbank Q. 2015;93(4):691–731. 10.1111/1468-0009.12162. 26626983 10.1111/1468-0009.12162 PMC4678937

[16] Kirk SFL, Ramos Salas X, Alberga AS, Russel-Mayhew S. Reducing Weight Bias in Obesity Management, Practice and Policy. In: Canadian Adult Obesity Practice Guidelines: Reducing Weight Bias in Obesity Management, Practice and Policy [Internet]. Available from: https://obesitycanada.ca/guidelines/weightbias/

[17] Lee MS, Gonzalez BD, Small BJ, Thompson JK. Internalized weight bias and psychological wellbeing: an exploratory investigation of a preliminary model. PLoS One. 2019;14(5):e0216324. 10.1371/journal.pone.0216324. 31071115 10.1371/journal.pone.0216324 PMC6508719

[18] Pearl RL, Puhl RM, Lessard LM, Himmelstein MS, Foster GD. Prevalence and correlates of weight bias internalization in weight management: a multinational study. SSM Popul Health. 2021;13:100755. 10.1016/j.ssmph.2021.100755. 33718581 10.1016/j.ssmph.2021.100755 PMC7920853

[19] Westbury S, Oyebode O, Van Rens T, Barber TM. Obesity stigma: causes, consequences, and potential solutions. Curr Obes Rep. 2023;12(1):10–23. 10.1007/s13679-023-00495-3. 36781624 10.1007/s13679-023-00495-3 PMC9985585

[20] Puhl RM, Lessard LM, Himmelstein MS, Foster GD. The roles of experienced and internalized weight stigma in healthcare experiences: perspectives of adults engaged in weight management across six countries. PLoS One. 2021;16(6):e0251566. 10.1371/journal.pone.0251566. 34061867 10.1371/journal.pone.0251566 PMC8168902

[21] Carter A, Steindl SR, Parker S, Gilbert P, Kirby JN. Compassion-focused therapy to reduce body weight shame for individuals with obesity: a randomized controlled trial. Behav Ther. 2023;54(5):747–64. 10.1016/j.beth.2023.02.001. 37597955 10.1016/j.beth.2023.02.001

[22] Pearl RL, Wadden TA, Jakicic JM. Is weight stigma associated with physical activity? A systematic review. Obesity. 2021;29(12):1994–2012. 10.1002/oby.23274. 34747131 10.1002/oby.23274 PMC8612947

[23] Bristow C, Meurer C, Simmonds J, Snell T. Anti-obesity public health messages and risk factors for disordered eating: a systematic review. Health Promot Int. 2020;35(6):1551–69. 10.1093/heapro/daaa018. 32150266 10.1093/heapro/daaa018

[24] Schmalz DL, Colistra CM. Obesity stigma as a barrier to healthy eating behavior. Top Clin Nutr. 2016;31(1):86–94. 10.1097/TIN.0000000000000060.

[25] Bidstrup H, Brennan L, Kaufmann L, De La Piedad Garcia X. Internalised weight stigma as a mediator of the relationship between experienced/perceived weight stigma and biopsychosocial outcomes: a systematic review. Int J Obes. 2022;46(1):1–9. 10.1038/s41366-021-00982-4. 10.1038/s41366-021-00982-4 PMC8501332 34628466

[26] Puhl RM, Himmelstein MS, Pearl RL. Weight stigma as a psychosocial contributor to obesity. Am Psychol. 2020;75(2):274–89. 10.1037/amp0000538. 32053000 10.1037/amp0000538

[27] Tomiyama AJ, Carr D, Granberg EM, Major B, Robinson E, Sutin AR, et al. How and why weight stigma drives the obesity ‘epidemic’ and harms health. BMC Med. 2018;16(1):123. 10.1186/s12916-018-1116-5. 30107800 10.1186/s12916-018-1116-5 PMC6092785

[28] Kalantzis MA, Maitland DM, Yannon M, Gaggiano C, He J, Barrita A, et al. Weight-based discrimination and cortisol output: a systematic review. Compr Psychoneuroendocrinol. 2025;22:100290. 10.1016/j.cpnec.2025.100290. 40297634 10.1016/j.cpnec.2025.100290 PMC12036022

[29] Hatzenbuehler ML, Phelan JC, Link BG. Stigma as a fundamental cause of population health inequalities. Am J Public Health. 2013;103(5):813–21. 10.2105/AJPH.2012.301069. 23488505 10.2105/AJPH.2012.301069 PMC3682466

[30] Campos-Vazquez RM, Gonzalez E. Obesity and hiring discrimination. Econ Hum Biol. 2020;37:100850. 10.1016/j.ehb.2020.100850. 31954211 10.1016/j.ehb.2020.100850

[31] Pearl RL, Hopkins CM, Bias. Stigma, and Social Consequences of Obesity. In: Kopelman PG, Caterson ID, Dietz WH, Armstrong S, Sweeting AN, Wilding JPH, editors. Clinical Obesity in Adults and Children [Internet]. 1st ed. Wiley; 2022 [cited 2026 Mar 24]. pp. 58–71. Available from: https://onlinelibrary.wiley.com/doi/10.1002/9781119695257.ch5 doi:10.1002/9781119695257.ch5.

[32] Puhl RM, Heuer CA. The stigma of obesity: a review and update. Obesity. 2009;17(5):941–64. 10.1038/oby.2008.636. 19165161 10.1038/oby.2008.636

[33] Himmelstein MS, Knepp KA, Phelan SM. The role of weight stigma in weight regain in bariatric surgery. Front Endocrinol. 2022;13:1076696. 10.3389/fendo.2022.1076696. 10.3389/fendo.2022.1076696 PMC9763922 36561565

[34] Homer CV, Tod AM, Thompson AR, Allmark P, Goyder E. Expectations and patients’ experiences of obesity prior to bariatric surgery: a qualitative study. BMJ Open. 2016;6(2):e009389. 10.1136/bmjopen-2015-009389. 26857104 10.1136/bmjopen-2015-009389 PMC4746450

[35] Mihaileanu FV, Fadgyas Stanculete M, Gherman C, Brata VD, Padureanu AM, Dita MO, et al. Beyond the physical: weight stigma and the bariatric patient journey. JCM. 2025;14(2):543. 10.3390/jcm14020543. 39860548 10.3390/jcm14020543 PMC11765684

[36] Raves DM, Brewis A, Trainer S, Han S, Wutich A. Bariatric surgery patients’ perceptions of weight-related stigma in healthcare settings impair post-surgery dietary adherence. Front Psychol. 2016;7:1497. 10.3389/fpsyg.2016.01497. 27777562 10.3389/fpsyg.2016.01497 PMC5056165

[37] Billing-Bullen G, Nielsen D, Wham C, Kruger R. Enablers and barriers to prevent weight-regain post bariatric surgery – a qualitative enquiry. Eating Behaviors. 2022;47:101677. 10.1016/j.eatbeh.2022.101677. 36252389 10.1016/j.eatbeh.2022.101677

[38] Vartanian LR, Fardouly J. The stigma of obesity surgery: negative evaluations based on weight loss history. OBES SURG. 2013;23(10):1545–50. 10.1007/s11695-013-0918-y. 23519633 10.1007/s11695-013-0918-y

[39] Bennett BL, Lawson JL, Funaro MC, Ivezaj V. Examining weight bias before and/or after bariatric surgery: a systematic review. Obes Rev. 2022. 10.1111/obr.13500. 36053042 10.1111/obr.13500

[40] Page MJ, McKenzie JE, Bossuyt PM, Boutron I, Hoffmann TC, Mulrow CD, et al. The PRISMA 2020 statement: an updated guideline for reporting systematic reviews. BMJ. 2021;n71. 10.1136/bmj.n71. 10.1136/bmj.n71 PMC8005924 33782057

[41] Harrison H, Griffin SJ, Kuhn I, Usher-Smith JA. Software tools to support title and abstract screening for systematic reviews in healthcare: an evaluation. BMC Med Res Methodol. 2020;20(1):7. 10.1186/s12874-020-0897-3. 31931747 10.1186/s12874-020-0897-3 PMC6958795

[42] Belur J, Tompson L, Thornton A, Simon M. Interrater reliability in systematic review methodology: exploring variation in coder decision-making. Sociological Methods & Research. 2021;50(2):837–65. 10.1177/0049124118799372.

[43] Wells G, Shea B, O’Connell D, Peterson J, Welch V, Losos M et al. Newcastle-Ottawa quality assessment scale cohort studies [Internet]. University of Ottawa; 2014. Available from: https://www.ncbi.nlm.nih.gov/books/NBK99082/bin/appb-fm4.pdf

[44] Sterne JAC, Savović J, Page MJ, Elbers RG, Blencowe NS, Boutron I, et al. RoB 2: a revised tool for assessing risk of bias in randomised trials. BMJ. 2019;l4898. 10.1136/bmj.l4898. 10.1136/bmj.l4898 31462531

[45] The Grade Working Group. GRADE [Internet]. Available from: https://www.gradeworkinggroup.org/

[46] Johanna Briggs Institute. Critical appraisal checklist for analytical cross sectional studies. 2016.

[47] Critical Appraisal Skills Programme. CASP Qualitative Studies Checklist [online] [Internet]. 2022. Available from: Available at: https://casp-uk.net/casp-tools-checklists/. Accessed: August 20th, 2023.

[48] Tolvanen L, Svensson Å, Hemmingsson E, Christenson A, Lagerros YT. Perceived and preferred social support in patients experiencing weight regain after bariatric surgery—a qualitative study. OBES SURG. 2021;31(3):1256–64. 10.1007/s11695-020-05128-5. 33205368 10.1007/s11695-020-05128-5 PMC7921025

[49] Feig EH, Amonoo HL, Onyeaka HK, Romero PM, Kim S, Huffman JC. Weight bias internalization and its association with health behaviour adherence after bariatric surgery. Clinical Obesity. 2020;10(4):e12361. 10.1111/cob.12361. 32319211 10.1111/cob.12361

[50] Lent MR, Napolitano MA, Wood GC, Argyropoulos G, Gerhard GS, Hayes S, et al. Internalized weight bias in weight-loss surgery patients: psychosocial correlates and weight loss outcomes. OBES SURG. 2014;24(12):2195–9. 10.1007/s11695-014-1455-z. 25337868 10.1007/s11695-014-1455-z

[51] Konings G, Drukker M, Severeijns R, Ponds R. The complexity of obesity-related health problems after bariatric surgery: the patient perspective. Obesity Pillars. 2023;7:100082. 10.1016/j.obpill.2023.100082. 37990685 10.1016/j.obpill.2023.100082 PMC10661984

[52] Han S, Agostini G, Brewis AA, Wutich A. Avoiding exercise mediates the effects of internalized and experienced weight stigma on physical activity in the years following bariatric surgery. BMC Obes. 2018;5(1):18. 10.1186/s40608-018-0195-3. 29988619 10.1186/s40608-018-0195-3 PMC6027738

[53] Hoffmann K, Paczkowska A, Bryl W, Marzec K, Raakow J, Pross M, et al. Comparison of perceived weight discrimination between Polish and German patients underwent bariatric surgery or endoscopic method versus conservative treatment for morbid obesity: an international multicenter study. Nutrients. 2022;14(13):2775. 10.3390/nu14132775. 35807955 10.3390/nu14132775 PMC9268827

[54] Garcia FK, Mulder BC, Wagemakers AMAE, Hazebroek EJ, Verkooijen KT. Understanding metabolic bariatric surgery stigma through the lived experiences of Dutch women. SSM Qual Res Health. 2026;9:100699. 10.1016/j.ssmqr.2026.100699.

[55] Pan A, Lucas M, Sun Q, Van Dam RM, Franco OH, Manson JAE, et al. Bidirectional association between depression and type 2 diabetes mellitus in women. Arch Intern Med. 2010;170(21):1884–91. 10.1001/archinternmed.2010.356. 21098346 10.1001/archinternmed.2010.356 PMC3065781

[56] Dunford A, Metzler A, Pittman B, Alperovich M, Price G, Ivezaj V. A prospective assessment of weight bias internalization in patients seeking body contouring after bariatric surgery. Surg Obes Relat Dis. 2025;21(12):1350–6. 10.1016/j.soard.2025.06.020. 40940272 10.1016/j.soard.2025.06.020

[57] McGarrity LA, Farnsworth HR, Aspinwall LG, Ibele AR, Terrill AL. Weight stigma and bariatric surgery: prospective improvements, psychological health, and weight. Health Psychol. 2025;44(10):936–43. 10.1037/hea0001517. 40471820 10.1037/hea0001517 PMC12353973

[58] Puhl R, Suh Y. Health consequences of weight stigma: implications for obesity prevention and treatment. Curr Obes Rep. 2015;4(2):182–90. 10.1007/s13679-015-0153-z. 26627213 10.1007/s13679-015-0153-z

[59] De Vries CEE, Terwee CB, Al Nawas M, Van Wagensveld BA, Janssen IMC, Liem RSL, et al. Outcomes of the first global multidisciplinary consensus meeting including persons living with obesity to standardize patient-reported outcome measurement in obesity treatment research. Obes Rev. 2022;23(8):e13452. 10.1111/obr.13452. 35644939 10.1111/obr.13452 PMC9539945

[60] Carels RA, Byrd R, Mansour L, Metzler AL, Jansen E. The role of weight stigma and internalized weight bias in health care avoidance and mistrust. Stigma Health. 2024 Oct;31. 10.1037/sah0000578.

[61] Phelan SM, Burgess DJ, Yeazel MW, Hellerstedt WL, Griffin JM, Ryn M. Impact of weight bias and stigma on quality of care and outcomes for patients with obesity. Obes Rev. 2015;16(4):319–26. 10.1111/obr.12266. 25752756 10.1111/obr.12266 PMC4381543

[62] Pearl RL, Wadden TA, Bach C, LaFata EM, Gautam S, Leonard S, et al. Long-term effects of an internalized weight stigma intervention: a randomized controlled trial. J Consult Clin Psychol. 2023;91(7):398–410. 10.1037/ccp0000819. 37155264 10.1037/ccp0000819

[63] Salas XR. Closing obesity care gaps and achieving health equity for people living with obesity. Eur J Intern Med. 2021;91:1–2. 10.1016/j.ejim.2021.06.016. 34226117 10.1016/j.ejim.2021.06.016

[64] Durso LE, Latner JD. Understanding self-directed stigma: development of the Weight Bias Internalization Scale. Obesity. 2008;16(S2):S80-6. 10.1038/oby.2008.448. 18978768 10.1038/oby.2008.448

