# Supplementary material for: Impact of Weight Bias, Stigma and Discrimination on Physical, Mental, and Quality of Life Outcomes of Metabolic and Bariatric Surgery: A Systematic Review
Source: Obes Surg. 2026 May 2;36(6):3338–49. doi: 10.1007/s11695-026-08696-0 (PMC13249764; doi:10.1007/s11695-026-08696-0)
Supplement: Supplementary file 1 — Supplementary Material 1 (DOCX 17.7 KB) [file 11695_2026_8696_MOESM1_ESM.docx]

**SUPPLEMENTAL MATERIAL – SEARCH STRATEGY (repeated again on February 22^nd^ 2024, September 3^rd^ 2025, and March 24^th^ 2026)**

**18 AUGUST 2023**

**Interface - EBSCOhost Research Databases**

**Search Screen - Basic Search**

**Database - CINAHL Complete : 203 RECORDS**

“obesity" OR "bariatric surgery" OR "metabolic surgery" OR "weight loss surgery" OR "gastric bypass" OR "RYGB" OR "gastric sleeve" OR "sleeve gastrectomy" OR "gastric banding" OR "duodenal switch" OR "SADI-S" OR "single anastomosis duodeno-ileal bypass with sleeve gastrectomy" OR "one anastomosis gastric bypass" OR "OAGB" OR "biliopancreatic diversion”

"coping strategies" OR "self-esteem" OR "patient engagement" OR "antisocial behavior" OR "exercise avoidance" OR "quality of life" OR "body image" OR "social relationships" OR "social life" OR "return to work" OR "return to education" OR "job stress" OR "job satisfaction" OR "Well- Being" OR "RTW" OR "Subjective well-being" OR "Life Satisfaction" OR "QOL"

"internalized weight bias" OR "self-stigma" OR "internalized stigma" OR "internalised weight stigma" OR "internalised weight discrimination" OR "weight-related stigma" OR "perceived weight stigma" OR "internalized stigma" OR "weight stigmatisation" OR "weight stigma concerns" OR "weight discrimination" OR "weight bias" OR "weight stigma"

**PUBMED search: 9th August 2023 : 31 records**

(("obesity" OR "bariatric surgery" OR "metabolic surgery" OR "weight loss surgery" OR "gastric bypass" OR "RYGB" OR "gastric sleeve" OR "sleeve gastrectomy" OR "gastric banding" OR "duodenal switch" OR "SADI-S" OR "single anastomosis duodeno-ileal bypass with sleeve gastrectomy" OR "one anastomosis gastric bypass" OR "OAGB" OR "biliopancreatic diversion") AND ("coping strategies" OR "self-esteem" OR "patient engagement" OR "antisocial behavior" OR "exercise avoidance" OR "quality of life" OR "body image" OR "social relationships" OR "social life" OR "return to work" OR "return to education" OR "job stress" OR "job satisfaction" OR "Well- Being" OR "RTW" OR "Subjective well-being" OR "Life Satisfaction" OR "QOL")) AND ("internalized weight bias" OR "self-stigma" OR "internalized stigma" OR "internalised weight stigma" OR "internalised weight discrimination" OR "weight-related stigma" OR "perceived weight stigma" OR "internalized stigma" OR "weight stigmatisation" OR "weight stigma concerns" OR "weight discrimination" OR "weight bias" OR "weight stigma") Filters: Clinical Trial, Comparative Study, Randomized Controlled Trial

**Cochrane REVIEW:**

**9th August 2023: 53 RECORDS**

**Search Name: weight stigma**

**Date Run: 09/08/2023 19:45:18**

**Comment:**

**ID Search Hits**

**#1** “obesity" OR "bariatric surgery" OR "metabolic surgery" OR "weight loss surgery" OR "gastric bypass" OR "RYGB" OR "gastric sleeve" OR "sleeve gastrectomy" OR "gastric banding" OR "duodenal switch" OR "SADI-S" OR "single anastomosis duodeno-ileal bypass with sleeve gastrectomy" OR "one anastomosis gastric bypass" OR "OAGB" OR "biliopancreatic diversion” 52311

**#2** "coping strategies" OR "self-esteem" OR "patient engagement" OR "antisocial behavior" OR "exercise avoidance" OR "quality of life" OR "body image" OR "social relationships" OR "social life" OR "return to work" OR "return to education" OR "job stress" OR "job satisfaction" OR "Well- Being" OR "RTW" OR "Subjective well-being" OR "Life Satisfaction" OR "QOL" 179070

**#3** "internalized weight bias" OR "self-stigma" OR "internalized stigma" OR "internalised weight stigma" OR "internalised weight discrimination" OR "weight-related stigma" OR "perceived weight stigma" OR "internalized stigma" OR "weight stigmatisation" OR "weight stigma concerns" OR "weight discrimination" OR "weight bias" OR "weight stigma" 546

**#4** #1 and #2 and #3 53

**Final PubMed Search**

**Date Run: 18 Aug 2023**

**Retrieved records: 415**

((coping strategies OR "self-esteem" OR "patient engagement" OR "antisocial behavior" OR "exercise avoidance" OR "body image" OR "social relationships" OR "return to work" OR "return to education" OR "job stress" OR "job satisfaction" OR "well-being" OR "RTW" OR "subjective well-being" OR "life satisfaction" OR "QOL" OR "stigma" OR "internalized weight bias" OR "self-stigma" OR "weight-related stigma" OR "internalized stigma" OR "mental health" OR "depression" OR "mood disorders" OR "anxiety disorders" OR "perceived stress" OR "substance use" OR "binge eating disorder" OR "eating disorders" OR "post-traumatic stress disorder" OR "perceived weight stigma")) AND (("bariatric surgery" OR "metabolic surgery" OR "sleeve gastrectomy" OR "gastric bypass" OR "RYGB" OR "RYGBP" OR "gastric banding" OR "biliopancreatic diversion" OR "duodenal switch" OR "BPD-DS" OR "single anastomosis duodeno-ileal bypass with sleeve gastrectomy" OR "one anastomosis gastric bypass" OR "OAGB" OR "SADI-S") AND ("clinical study"[Filter] OR "clinical trial"[Filter] OR "comparative study"[Filter] OR "controlled clinical trial"[Filter] OR "multicenter study"[Filter] OR "observational study"[Filter] OR "randomized controlled trial"[Filter]))
